# Admission laboratory parameters for early risk stratification after aneurysmal subarachnoid hemorrhage: a machine learning analysis of real-world data

**DOI:** 10.1016/j.bas.2026.106270

**Published:** 2026-08-07

**Authors:** Anton Früh, Nora Dengler, Cihat Karadag, Paul Pöser, Peter Vajkoczy, Stefan Wolf

**Affiliations:** a Department of Neurosurgery, Charité - Universitätsmedizin Berlin, Berlin, Germany, Charitéplatz 1, Berlin, 10117, Germany; b Faculty of Health Sciences Brandenburg, Medical School Theodor Fontane, Campus Bad Saarow, Germany; c Department of Neurosurgery, HELIOS Hospital Bad Saarow, Germany

**Keywords:** Subarachnoid hemorrhage, Neurological outcome, Biomarker, Admission, Laboratory, Machine learning

## Abstract

**Background:**

Aneurysmal subarachnoid hemorrhage (aSAH) is associated with substantial morbidity and mortality. Early risk stratification is essential for treatment planning and prognostication, yet neurological assessment may be limited in sedated or critically ill patients. Routine admission laboratory parameters may provide additional prognostic information and improve machine learning (ML)-based outcome prediction.

**Research question:**

To evaluate the predictive value of routinely obtained admission laboratory parameters for unfavorable neurological outcome after aSAH and to compare different ML algorithms.

**Material and methods:**

This retrospective analysis included prospectively collected data from 389 patients with aSAH. Unfavorable outcome was defined as modified Rankin Scale (mRS) > 2 at 6 months. Four feature sets were analyzed: laboratory values, clinical variables, radiographic features and a combined feature set. Logistic regression, random forest, XGBoost and support vector machine models were evaluated using 5-fold-stratified-cross-validation. Feature importance was assessed using SHAP analysis.

**Results:**

Laboratory-only models achieved moderate discrimination, with random forest demonstrating the highest performance (AUC = 0.724). Clinical models showed superior predictive accuracy overall (best AUC = 0.785), whereas radiographic-only models achieved lower performance. The combined feature set achieved the highest discrimination and outperformed the clinical-only model (p = 0.016), whereas laboratory-only and clinical-only discrimination did not differ significantly. The most important laboratory predictors were red blood cell distribution width, leukocyte count, glucose, and C-reactive protein.

**Discussion and conclusion:**

Routine admission laboratory parameters carry modest but incremental prognostic information beyond established clinical grading when integrated through non-linear ML, but they do not replace clinical assessment. These single-center findings are hypothesis-generating and require external validation before clinical use.

## Highlights

• Routine admission laboratory parameters add prognostic value beyond clinical grading after aneurysmal subarachnoid hemorrhage.
• Combining laboratory, clinical, and radiographic features improved machine learning-based outcome prediction performance.
• Red blood cell distribution width, leukocyte count, glucose, and C-reactive protein were the most important laboratory predictors.
• Laboratory data complement rather than replace neurological assessment.

## 1 Introduction

Aneurysmal subarachnoid hemorrhage (aSAH) is associated with substantial morbidity and mortality rates (Connolly et al., 2012). Accurate and reliable prediction of clinical outcomes after aSAH is important, given its influence on therapeutic decision-making processes, long-term care planning, and patient counseling (Jaja et al., 2018; Dengler et al., 2021).

Clinical grading scales, including the Hunt & Hess (HH) grade, World Federation of Neurosurgical Societies (WFNS) score and Glasgow Coma Scale (GCS) are widely used for the course prediction following aSAH. While these scores capture the neurological presentation at admission, they do not integrate the wealth of routine laboratory data available at the time of hospital admission. In recent years, the utilization of biomarkers to predict disease progression and clinical outcomes has become prevalent across a wide range of health conditions (Jaja et al., 2018). In this context, admission laboratory parameters may provide crucial predictive information. Recent evidence suggests that, during the course of in-hospital stay, changes in levels of biomarkers, such as creatinine, CRP, and glucose, are associated with clinical outcomes in aSAH (Connolly et al., 2012; Brouwers et al., 2012; Oertel et al., 2006; Vos et al., 2006; Lin et al., 2023). However, the predictive merit of these parameters at the time of admission remains elusive. The red blood cell distribution width (RDW) quantifies the variability of erythrocyte volumes and was identified as predictive marker for cerebrovascular diseases and traumatic brain injury (Li et al., 2017; Lin et al., 2023; Lorente et al., 2021). However, these analyses have typically been univariate or focused on a small number of selected biomarkers, without leveraging the full spectrum of available admission laboratory data.

Machine learning (ML) offers a methodological framework for integrating multiple, potentially correlated predictors into a unified prediction model. ML-based approaches have shown promise in neurosurgical outcome prediction and have been applied to aSAH in the context of vasospasm prediction, hydrocephalus risk, and mortality estimation (Frey et al., 2023; Palermo et al., 2025; de Jong et al., 2021). Given that early prediction of outcomes in aSAH on admission may be key in tailoring therapeutic strategies, our study aims to evaluate predictive value of admission laboratory data for neurological outcome, and to identify which laboratory parameters contribute most to model performance.

## 2 Materials and methods

### 2.1 Study design

This retrospective study was conducted at a tertiary academic medical center in a European capital providing advanced specialty care. The institution comprises two hospital campuses, where patients were treated by the same neurosurgical team. This study was approved by the local Ethics Committee (EA1/291/14) and is in accordance with the Declaration of Helsinki. The manuscript was prepared in accordance with the Transparent Reporting of a multivariable prediction model for Individual Prognosis or Diagnosis (TRIPOD) statement and its recent extension for models using machine learning (TRIPOD + AI) (Collins et al., 2015, 2024).

### 2.2 Data acquisition and outcome

Patients hospitalized for aSAH between January 1, 2009, and December 31, 2015 were included. The initial diagnosis of a SAH was confirmed by various methods described previously, comprising computed tomography, digital subtraction angiography, or the detection of xanthochromia in the cerebrospinal fluid using lumbar puncture (Frey et al., 2023). Only patients with a ruptured intracranial aneurysm confirmed by CT angiography and/or digital subtraction angiography were included in the present study. Patients were treated according to international guidelines for treatment of aSAH (Dengler et al., 2021; Steiner et al., 2013; Sandow et al., 2016; Diesing et al., 2018). Primary outcomes were quantified according to two groups: a) Modified Rankin Scale (mRS) (van Swieten et al., 1988) > 2 representing unfavorable outcome, and b) mRS ≤ 2 representing favorable outcomes. Follow-up mRS was obtained from files of scheduled control visits or systematic telephone interviews 6 months after aSAH. In-hospital mortality was evaluated using the clinical database.

### 2.3 Laboratory and combined feature sets

Four feature configurations were evaluated: (i) a laboratory set comprising 21 routinely obtained admission parameters: red cell distribution width (RDW), C-reactive protein (CRP), glucose, creatinine, creatine kinase (CK), CK-MB, troponin T, international normalized ratio (INR), partial thromboplastin time (pTT), thyroid-stimulating hormone (TSH), hemoglobin, hematocrit, erythrocyte count, leukocyte count, platelet count, mean corpuscular volume, mean corpuscular hemoglobin, mean corpuscular hemoglobin concentration, mean platelet volume, sodium, and lactate. (ii) a clinical set including neurological severity and demographic variables (GCS, WFNS score, Hunt & Hess grade, age, BMI and sex). (iii) a radiographic set incorporating imaging-derived features (modified and original Fisher grade, BNI score, presence of intraparenchymal or intraventricular hemorrhage and midline shift). (iv) a combined set integrating all variables. Missing values were handled by column-wise median imputation within each cross-validation fold to prevent data leakage. Missingness rates varied widely across lab parameters, from < 2% (INR, creatinine) to 63.5% (lactate) and 71.7% (CK-MB), reflecting real-world clinical practice where not all labs are obtained for every patient. No patients were excluded due to missing laboratory data.

### 2.4 Models and evaluation

Four classifiers were specified a priori and selected to represent complementary model families, thereby reducing dependence on a single inductive bias. The following supervised classifiers were trained: L2-regularized logistic regression with feature standardization using StandardScaler as an interpretable linear reference (Collins et al., 2015), random forest with 500 estimators, to capture nonlinear relationships and feature interactions (Breiman, 2001), XGBoost with 300 estimators and a learning rate of 0.1 as a regularized gradient-boosting method for structured tabular data (Chen and Guestrin, 2016), and support vector machines with a radial basis function kernel as a margin-based non-linear classifier (Cortes and Vapnik, 1995). Hyperparameters were fixed a priori. Model performance was assessed using 5-fold stratified cross-validation. Evaluation metrics included AUC-ROC, sensitivity, specificity, F1-score, and Brier score. SHAP analysis was applied to random forest models to assess feature-specific contributions (Ali et al., 2023). Random forest feature importance was additionally quantified using the mean decrease in Gini impurity, reflecting the reduction in node heterogeneity achieved by each feature across the ensemble. Pairwise differences in AUC between feature sets were tested using the paired bootstrap. Calibration was evaluated using the Hosmer–Lemeshow test and calibration plots. Three robustness analyses were pre-specified. First, sensitivity analyses repeated the models after excluding laboratory parameters with > 30% and > 50% missingness. Second, a sequential-entry hold-out analysis trained models on the first 70% of consecutively enrolled patients and tested them on the last 30%, serving as a proxy for temporal validation. Third, the incremental value of laboratory data was benchmarked against a SAHIT-type clinical reference model comprising age, WFNS grade, modified Fisher grade, aneurysm size, aneurysm location, and treatment modality. The analyses were conducted in Python 3.12 (scikit-learn v1.3, XGBoost v2.0, shap v0.44).

## 3 Results

### 3.1 Patient characteristics

A total of 389 patients were included in the study, with a median age of 54 years (IQR 46–63). The cohort comprised 70.4% female patients. Favorable outcomes were observed in 171 patients (43.9%), while 218 (56.1%) exhibited unfavorable outcomes. Baseline characteristics stratified by outcome groups are presented in Table 1. The admission laboratory values are summarized in Table 2.

**Table 1:** **Baseline clinical characteristics stratified by neurological outcome (n=389).** Values are median (IQR) or n (%). p-values from Mann–Whitney *U* test (continuous variables) or chi-squared test (categorical variables). Abbreviations: GCS = Glasgow Coma Scale; WFNS = World Federation of Neurosurgical Societies; IQR = interquartile range.

Variable	Favorable outcome (n = 171)	Unfavorable outcome (n = 218)	p-value	Missing, n (%)
**Age and Sex**				
Age, years, median (IQR)	51.0 (44.0–59.0)	56.0 (48.0–66.0)	**<0.001**	0 (0.0%)
Female sex, n (%)	115 (67.3%)	159 (72.9%)	0.268	0 (0.0%)
Body mass index, kg/m^2^, median (IQR)	25.1 (23.1–27.2)	24.8 (22.5–27.8)	0.698	67 (17.2%)
**Glasgow Coma Scale**				
GCS, median (IQR)	15.0 (13.0–15.0)	5.0 (3.0–13.0)	**<0.001**	0 (0.0%)
**WFNS Grade, n (%)**			**<0.001**	0 (0.0%)
Grade I	103 (60.2%)	40 (18.3%)		
Grade II	21 (12.3%)	17 (7.8%)		
Grade III	4 (2.3%)	9 (4.1%)		
Grade IV	15 (8.8%)	34 (15.6%)		
Grade V	28 (16.4%)	118 (54.1%)		
**Hunt & Hess Grade, n (%)**			**<0.001**	0 (0.0%)
Grade I	67 (39.2%)	27 (12.4%)		
Grade II	46 (26.9%)	22 (10.1%)		
Grade III	26 (15.2%)	32 (14.7%)		
Grade IV	15 (8.8%)	40 (18.3%)		
Grade V	17 (9.9%)	97 (44.5%)		
**Localization of Aneurysm in the posterior circulation, n (%)**	26 (15.2%)	43 (19.7%)	0.305	0 (0.0%)
Modified Fisher-Score, **n (%)**			**<0.001**	0 (0.0%)
Grade 0	13 (7.6%)	5 (2.3%)		
Grade 1	30 (17.5%)	18 (8.3%)		
Grade 2	12 (7.0%)	11 (5.0%)		
Grade 3	60 (35.1%)	44 (20.2%)		
Grade 4	56 (32.7%)	140 (64.2%)

**Table 2:** **Admission laboratory values stratified by neurological outcome (n=389).** Values are median (IQR). p-values from Mann–Whitney *U* test. Bold p-values indicate statistical significance (p < 0.05). Favorable outcome = mRS 0-2. Abbreviations: RDW = red cell distribution width, CRP = C-reactive protein, CK = creatine kinase, pTT = partial thromboplastin time, MCV = mean corpuscular volume, MCH = mean corpuscular hemoglobin, MCHC = mean corpuscular hemoglobin concentration, MPV = mean platelet volume, TSH = thyroid-stimulating hormone, IQR = interquartile range.

Laboratory Parameter	Favorable outcome (n = 171)	Unfavorable outcome (n = 218)	p-value	Missing n (%)
**Inflammatory & Metabolic Markers**				
CRP (mg/l)	0.79 (0.30–2.12)	1.31 (0.31–5.49)	**0.014**	71 (18.3%)
Glucose (mg/dl)	129 (110–152)	153 (128–193)	**< 0.001**	135 (34.7%)
Leukocytes (10^9^/l)	11.5 (8.8–16.5)	11.7 (8.8–15.0)	0.610	9 (2.3%)
**Renal & Cardiac Markers**				
Creatinine (mg/dl)	0.73 (0.61–0.88)	0.78 (0.64–0.96)	**0.017**	7 (1.8%)
CK (U/l)	101 (68–159)	113 (70–196)	0.351	103 (26.5%)
CK-MB (U/l)	18 (14–31)	25 (14–42)	0.186	279 (71.7%)
Troponin T (ng/l)	0.0 (0.0–9.2)	0.1 (0.0–11.2)	0.313	215 (55.3%)
**Coagulation**				
INR	1.05 (1.00–1.09)	1.06 (1.00–1.13)	0.077	6 (1.5%)
pTT (sec)	30.6 (28.4–33.3)	31.3 (28.6–33.7)	0.709	9 (2.3%)
**Complete Blood Count**				
Hemoglobin (g/dl)	12.90 (11.90–14.00)	13.10 (11.90–14.20)	0.480	97 (24.9%)
Hematocrit (l/l)	0.38 (0.35–0.42)	0.39 (0.35–0.42)	0.484	101 (26.0%)
Erythrocytes (10^12^/l)	4.30 (3.90–4.70)	4.30 (3.86–4.70)	0.898	99 (25.4%)
Thrombocytes (10^9^/l)	228 (198–284)	224 (193–265)	0.450	99 (25.4%)
MCV (fl)	89.0 (85.8–92.0)	90.0 (87.0–93.0)	0.055	100 (25.7%)
MCH (pg)	30.40 (29.35–31.52)	30.70 (29.60–31.60)	0.381	100 (25.7%)
MCHC (g/dl)	34.00 (33.40–34.75)	33.85 (33.10–34.80)	0.347	102 (26.2%)
MPV (fl)	10.30 (10.00–11.00)	10.25 (10.00–11.00)	0.843	102 (26.2%)
RDW (%)	13.10 (12.70–13.50)	13.80 (13.05–14.45)	**< 0.001**	39 (10.0%)
**Endocrine & Other**				
TSH (mIU/l)	1.94 (1.06–3.06)	1.95 (0.93–3.72)	0.775	180 (46.3%)
Sodium (mmol/l)	140.0 (137.5–143.0)	139.0 (137.0–141.0)	0.275	230 (59.1%)
Lactate (mmol/l)	13 (8–18)	16 (10–24)	0.092	247 (63.5%)

### 3.2 Model performance

**Fig. 1:**
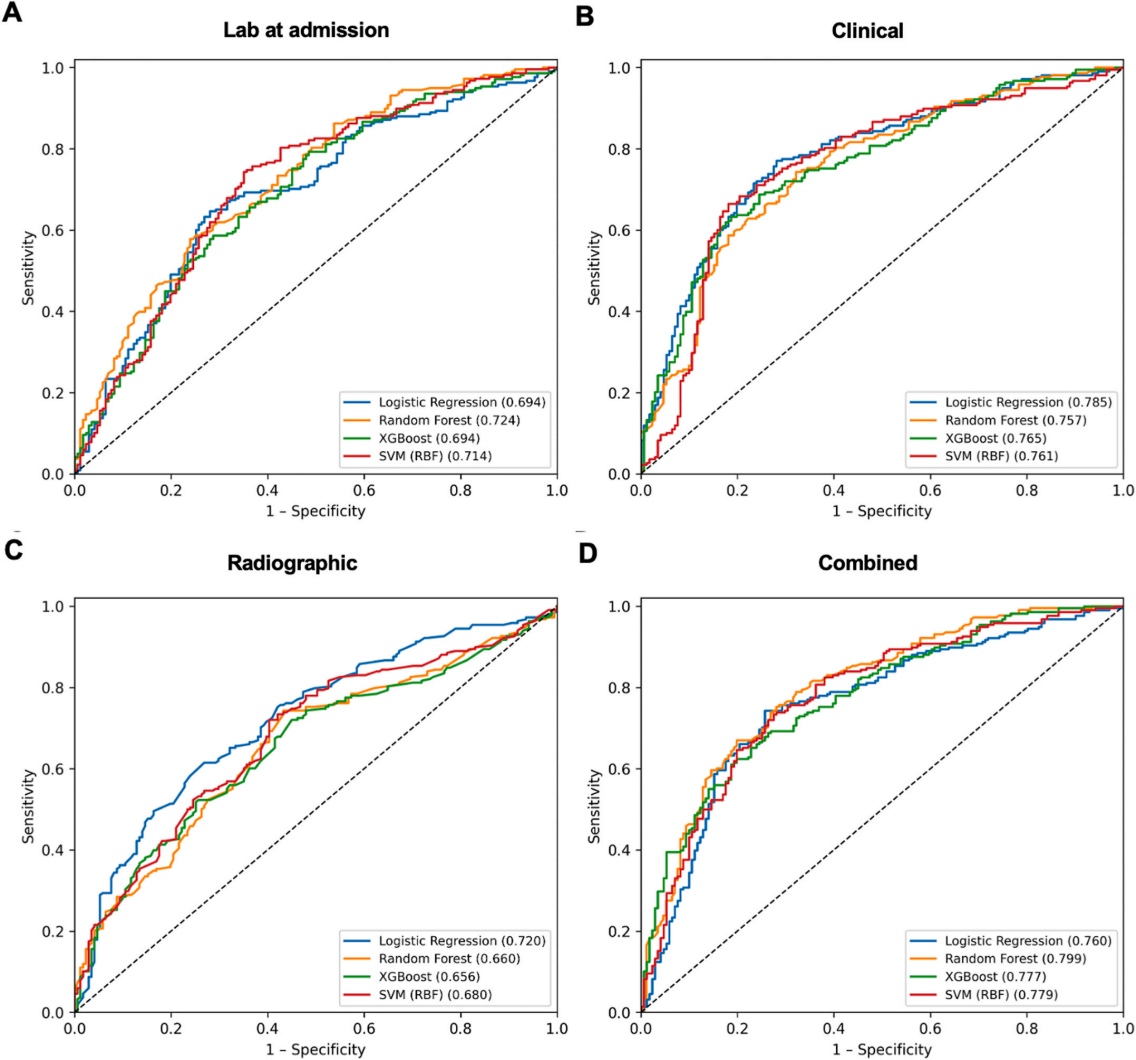
**ROC curves for ML-based prediction of unfavorable neurological outcome after aSAH across different feature sets.** (A) Laboratory parameters at admission, (B) clinical features (C) radiographic features, and (D) combined feature set integrating all variables. Model performance is shown for logistic regression (blue), random forest (orange), XGBoost (green), and support vector machine with radial basis function kernel (red). The dashed diagonal line represents chance-level discrimination (AUC = 0.5). Abbreviations: AUC = Area under the curve, ROC = Receiver operating characteristic.

**Fig. 2:**
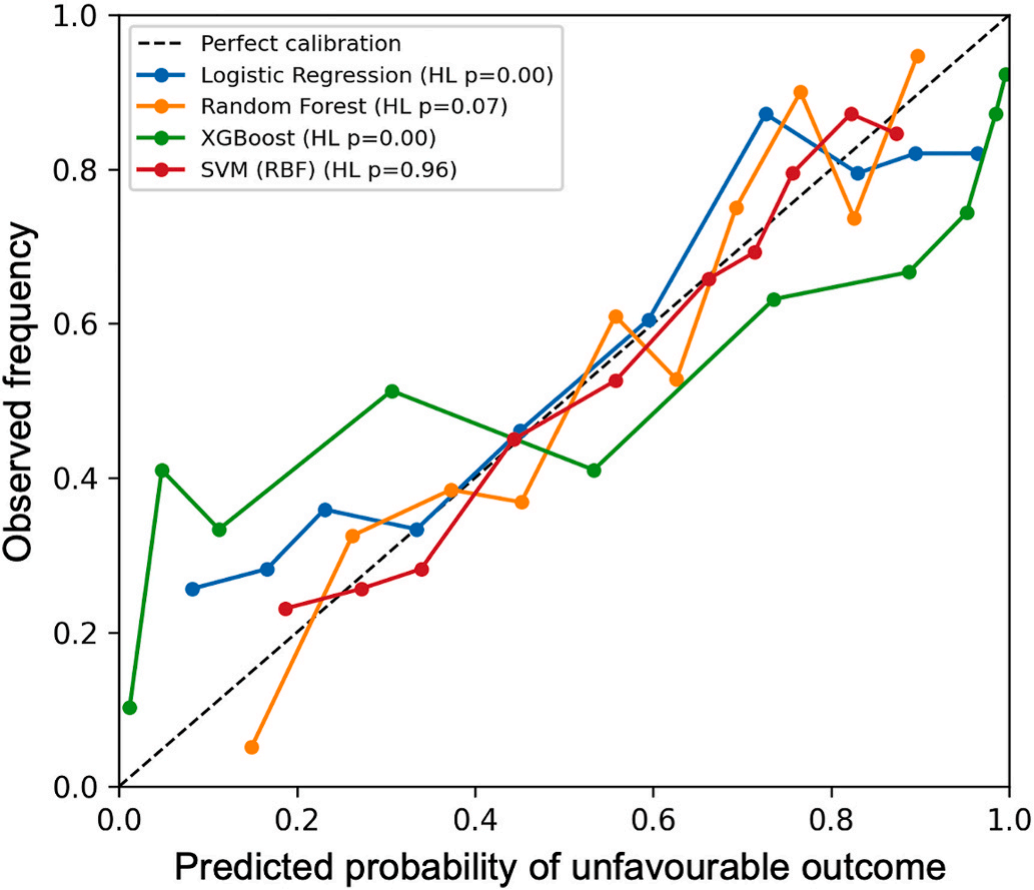
**Calibration plots for the four combined-set models with Hosmer-Lemeshow values.** Abbreviations: HL = Hosmer-Lemeshow p-value.

The results of the ML model performance across all four feature sets are presented in Fig. 1 and Table 3. Laboratory-only models achieved moderate discrimination, with random forest performing best (AUC 0.724, 95%-CI 0.670 - 0.772). Clinical-only models discriminated best (logistic regression AUC 0.785, 95%-CI 0.736 - 0.828), whereas radiographic-only models performed the weakest (AUC 0.66 - 0.72). The combined model achieved the highest discrimination (random forest AUC 0.799, 95%-CI 0.753 - 0.842, support vector machine 0.779, 95%-CI 0.730-0.824). Differences between the feature sets were tested by paired bootstrap. The combined model outperformed both the laboratory-only model (random forest ΔAUC +0.074, 95% CI + 0.030 to +0.118, p < 0.001) and the clinical-only model (random forest ΔAUC +0.043, +0.007 to +0.078, p = 0.016), indicating that laboratory data contributed information beyond clinical grading. In contrast, the analysis between laboratory-only and clinical-only discrimination showed no difference for the random forest analysis (ΔAUC +0.031, −0.027 to +0.091, p = 0.32), indicating that routinely available laboratory panel alone approached the performance of established clinical scores. Excluding laboratory parameters with high missingness had negligible impact on combined model discrimination (random forest AUC = 0.799 for all 21 parameters, AUC = 0.802 excluding parameters > 50% missing, and AUC = 0.800 excluding parameters > 30% missing). The data is presented in Supplementary Table S1. Calibration of the combined models is shown in Fig. 2. The random forest and support vector machine models were well calibrated (Hosmer–Lemeshow p = 0.07 and 0.96), whereas the reweighted logistic regression and XGBoost models showed miscalibration (p < 0.001). This may reflect the effect of class weighting and boosting on predicted-probability scale. Probability recalibration would therefore be advisable before deployment of these two models.

**Table 3:** **ML Model Performance for the prediction of neurological outcome.** *Best AUC per feature set in ROC-Analysis. ^#^Best sensitivity/specificity balance. Abbreviations: AUC = Area under the curve, CI = Confidence Interval, NPV = negative predictive value, PPV = positive predictive value.

Feature Set	Model	AUC (95%-CI)	Brier Score	Sensitivity	Specificity	PPV	NPV	F1-Score
**Lab at Admission**	Random Forest*	**0.724 (0.670-0.772)**	**0.208**	**57.8%**	**76.0%**	**75.4%**	**58.6%**	**0.655**
	SVM	0.714 (0.659-0.766)	0.212	74.3%	64.9%	73.0%	66.5%	0.736
	Logistic Reg.^#^	0.694 (0.641-0.744)	0.230	64.7%	71.9%	74.6%	61.5%	0.693
	XGBoost	0.694 (0.637-0.746)	0.256	79.4%	51.5%	67.6%	66.2%	0.730
**Clinical**	Logistic Reg.*^,#^	**0.785 (0.736-0.828)**	**0.187**	**77.1%**	**71.9%**	**77.8%**	**71.1%**	**0.774**
	XGBoost	0.765 (0.712-0.810)	0.210	63.3%	81.3%	81.2%	63.5%	0.711
	SVM	0.761 (0.707-0.808)	0.188	66.5%	81.9%	82.4%	65.7%	0.736
	Random Forest	0.757 (0.707-0.803)	0.204	74.3%	67.8%	74.7%	67.4%	0.745
**Radiographic**	Logistic Reg. *^,#^	**0.720 (0.667-0.771)**	**0.215**	**61.5%**	**73.1%**	**74.4%**	**59.8%**	**0.673**
	SVM	0.680 (0.624-0.730)	0.223	72.0%	59.6%	69.5%	62.6%	0.707
	XGBoost	0.660 (0.604-0.713)	0.242	74.3%	56.7%	68.6%	63.4%	0.714
	Random Forest	0.656 (0.600-0.711)	0.239	72.0%	55.0%	67.1%	60.6%	0.695
**Combined**	Random Forest *	**0.799 (0.753-0.842)**	**0.180**	**75.2%**	**71.9%**	**77.4%**	**69.5%**	**0.763**
	SVM	0.779 (0.730-0.824)	0.188	73.4%	72.5%	77.3%	68.1%	0.753
	XGBoost	0.777 (0.731-0.822)	0.223	62.4%	80.1%	80.0%	62.6%	0.701
	Logistic Reg.^#^	0.760 (0.708-0.807)	0.201	74.3%	74.3%	78.6%	69.4%	0.764

### 3.3 Sequential-entry validation and benchmarking

In a sequential-entry analysis, discrimination was retained in the later-enrolled test patients (combined random forest test AUC 0.754, 95% CI 0.664-0.842, laboratory 0.720, clinical 0.675-0.735). The data is presented in Supplementary Table S2. Benchmarked against a SAHIT-type clinical reference model adding the admission laboratory panel significantly improved discrimination for the random forest (AUC 0.718 to 0.797, ΔAUC +0.079, +0.037 to +0.119, p < 0.001) but not for logistic regression (ΔAUC -0.010, p = 0.52), indicating that the incremental information carried by laboratory data is realized primarily through non-linear feature interactions. The corresponding data is presented in Supplementary Table S3.

### 3.4 Feature importance and SHAP analysis

**Fig. 3:**
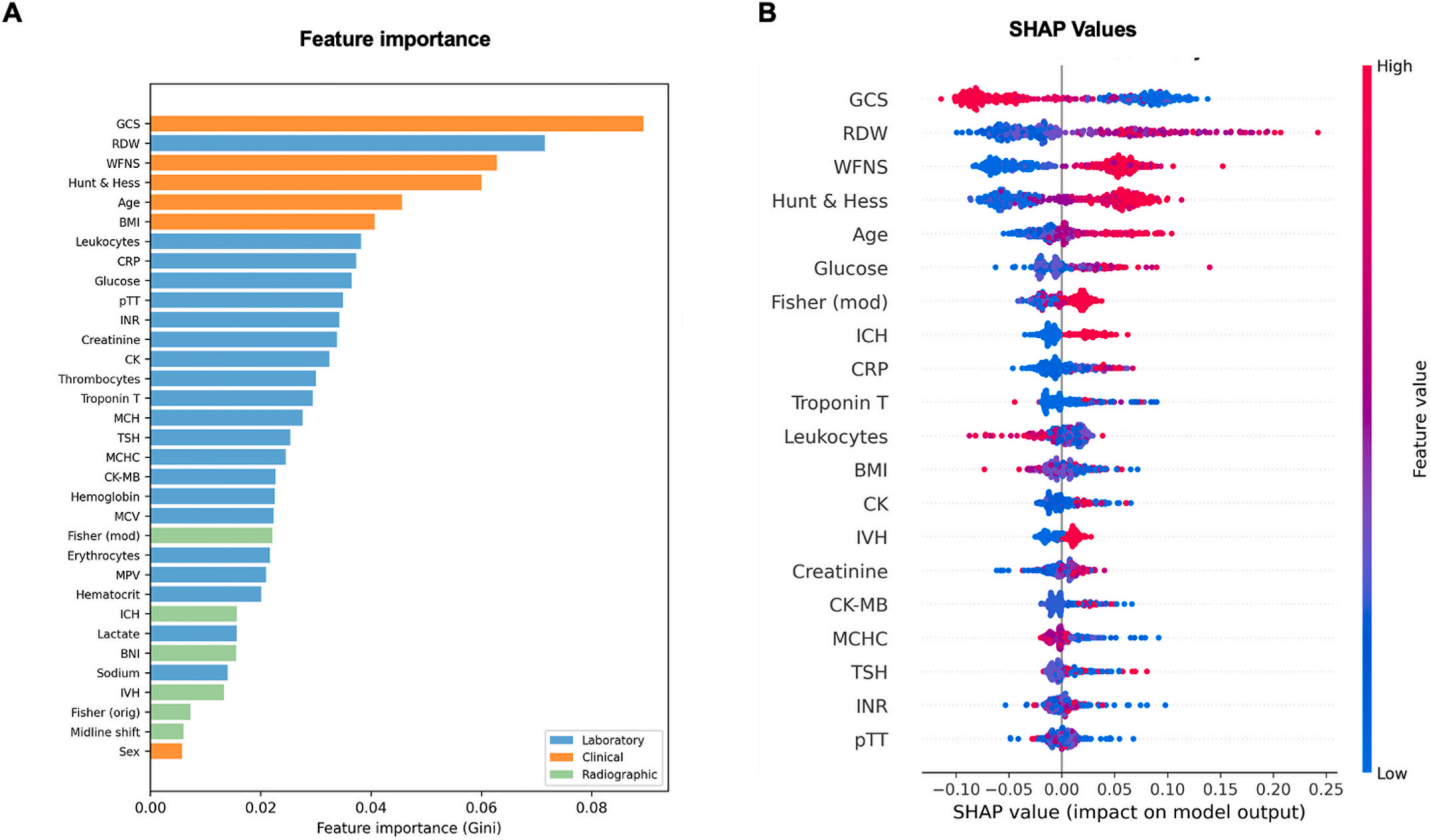
**Random Forest Feature importance and SHAP-based interpretability analysis of the combined model**. (A) Global feature importance derived from the random forest model, ranked by mean decrease in Gini impurity. Clinical variables (orange), laboratory parameters at admission (blue), and radiographic features (green) are shown. (B) SHAP (SHapley Additive exPlanations) summary plot illustrating the impact of individual features on model output. Each point represents a single patient, with color indicating the feature value (blue = low, red = high).

**Fig. 4:**
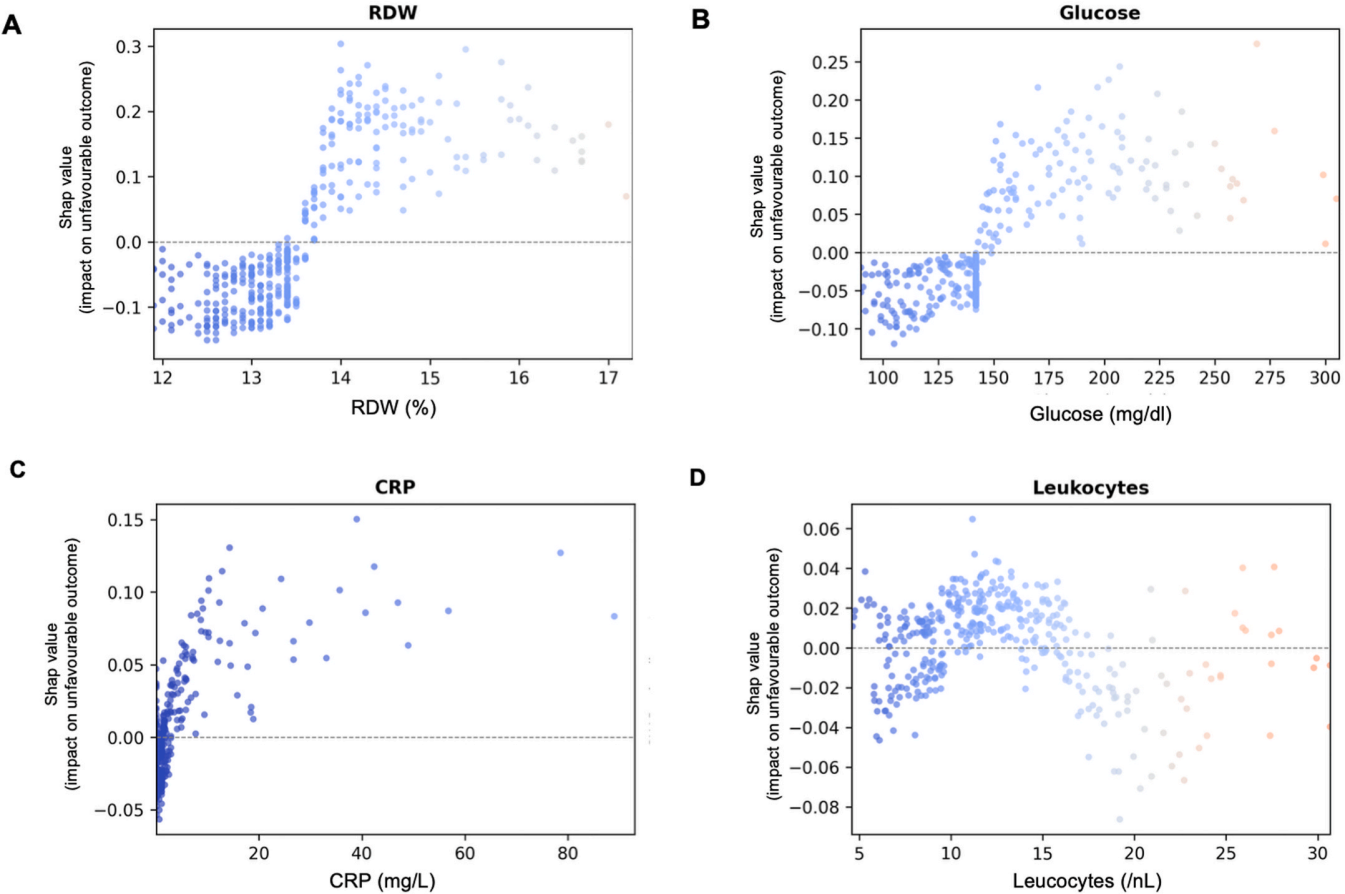
SHAP dependence plots for the top laboratory predictors in the laboratory-only random-forest model. A. red cell distribution width. B. glucose, C. C-reactive protein, D. leukocyte count.

The Random Forest feature importance analysis for the combined model is shown in Fig. 3. The clinical scores dominated the top rankings. The most important lab-based predictors were RDW (importance = 0.072), leucocytes (importance = 0.038), glucose (importance = 0.037) and CRP (importance = 0.037). The SHAP dependence analysis of the top performing lab values in the laboratory-only model is shown in Fig. 4. The data demonstrate threshold-shaped associations. The risk contribution of RDW increased sharply above approximately 13.5-14%, and that of glucose above approximately 140-150 mg/dL, while higher CRP was associated with a graded increase in predicted risk.

### 3.5 Prediction of in-hospital mortality

**Fig. 5:**
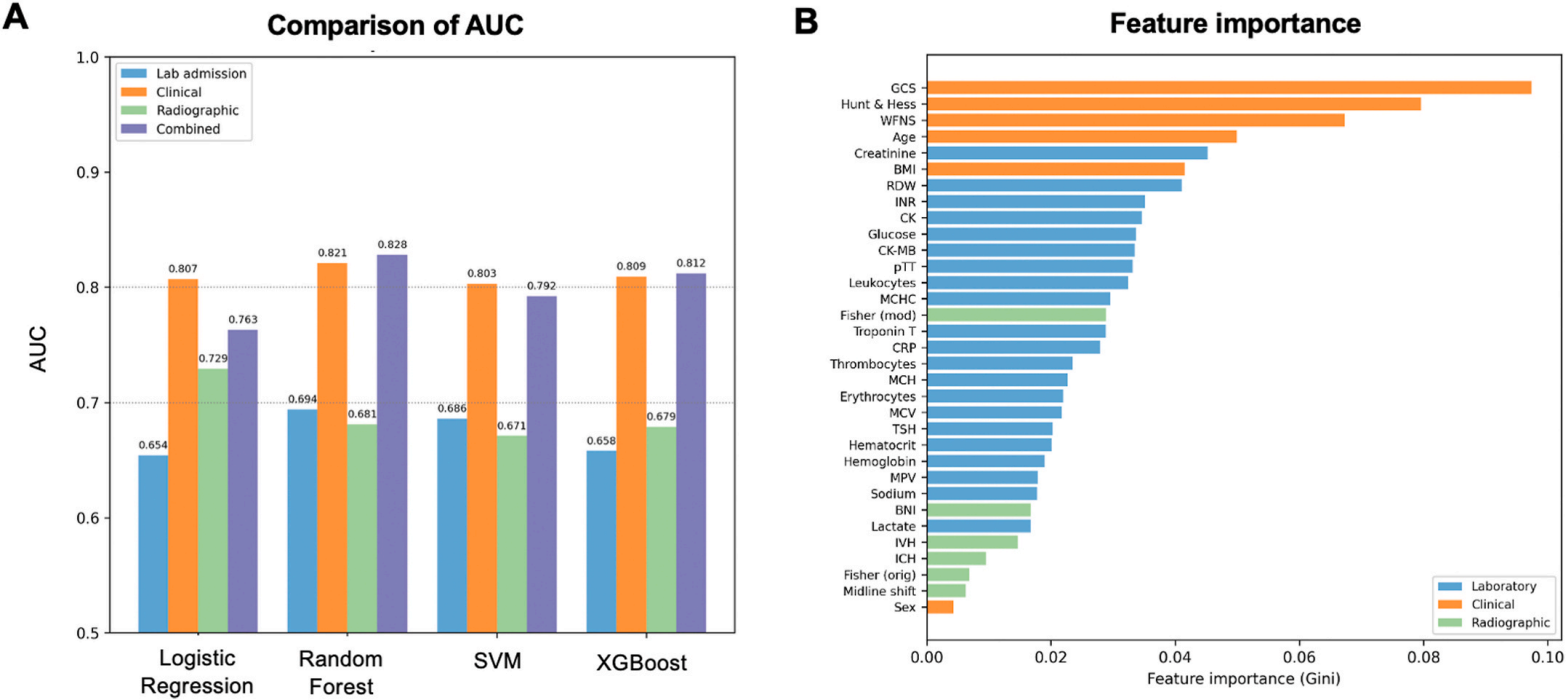
**Model performance comparison and feature importance for the prediction of mortality.** (A) Comparison of predictive performance across feature sets (laboratory at admission, clinical, radiographic, and combined) for four classifiers: logistic regression, random forest, support vector machine (SVM), and XGBoost. Model performance is expressed as area under the receiver operating characteristic curve (AUC). (B) Global feature importance derived from the random forest model based on the combined feature set, ranked by mean decrease in Gini impurity. Clinical variables (orange), laboratory parameters (blue), and radiographic features (green) are shown.

In an exploratory analysis of in-hospital mortality (107 deaths, 27.5%), clinical variables were the principal contributors. The clinical feature set alone achieved an AUC of 0.821 (95%-CI 0.773-0.867, random forest model), whereas the laboratory set alone was weaker (AUC = 0.694, 95%-CI 0.634-0.753). In contrast to the neurological outcome, adding laboratory and radiographic data to the clinical set did not improve discrimination (combined AUC 0.828, 0.780-0.871, ΔAUC +0.006, 95% CI -0.029 to 0.042, p = 0.76), indicating that the incremental value of admission laboratories is outcome-specific. Negative predictive value was consistently high (88-94%) while positive predictive value remained modest (37-55%). The detailed results are provided in Fig. 5 and Supplementary Table S4.

## 4 Discussion

The principal finding of this study is that routinely obtained admission laboratory parameters provide clinically relevant information for early risk stratification in patients with aSAH. Although clinical severity scores remained the strongest individual predictors, laboratory values alone achieved meaningful discrimination of unfavorable neurological outcome and improved model performance when combined with established clinical and radiographic features. These findings support the complementary value of routinely available laboratory data in multimodal prognostic assessment at presentation.

The present data demonstrate that routine admission laboratory values alone provide clinically meaningful discrimination of neurological outcome with the best performance achieved using Random Forest Algorithms. This is notable, as these data are universally available, objective, and obtained as part of standard clinical care, without requiring clinical scoring, neurological examination, or physician assessment. Our observation regarding elevated serum creatinine levels aligns with prior research on the impact of renal dysfunction on the prognosis of aSAH (Lampmann et al., 2022; Zacharia et al., 2009). Acute kidney injury is a frequent phenomenon in aSAH patients due to hypovolemia, neurogenic pulmonary edema, or administration of contrast agents, which in turn may complicate the clinical course and worsen outcomes. The findings of this study align with those reported by Lampmann et al. (2022), demonstrating that elevated serum creatinine levels at admission are linked to adverse neurological outcomes after a six-month period. Increased serum CRP levels found in our cohort support the growing body of evidence that emphasizes the role of systemic inflammation in the progression and prognosis of aSAH (McMahon et al., 2013). CRP, a widely accepted marker of inflammation (Sproston and Ashworth, 2018), can reflect the extent of inflammatory responses triggered by aSAH, which may contribute to secondary brain injury and poor clinical outcomes. Additionally, our findings indicate a negative impact of elevated glucose levels on outcomes after aSAH. This is in line with Badjatia et al. (2005) showing a correlation between hyperglycemia and the occurrence of vasospasms after aSAH. A study conducted by Frontera et al. (2006) disclosed that heightened serum glucose levels, measured within the first three days following subarachnoid hemorrhage (SAH), were correlated with prolonged stays in intensive care units and unfavorable outcomes. The frequent occurrence of high admission levels of serum glucose and their association with poor neurological outcomes were further corroborated by a meta-analysis (Kruyt et al., 2009). Among the evaluated biomarkers, RDW emerged as the strongest individual laboratory predictor. RDW has been associated with systemic inflammation, oxidative stress, and reduced physiological reserve, all of which may contribute to secondary brain injury after aSAH. Its prognostic relevance has been demonstrated in stroke and critical care populations, supporting its role as a readily available marker of global physiological stress (Shen and Shen, 2024; Zhang et al., 2025). Notably, laboratory-only models achieved performance levels approaching those of clinical models, suggesting that routinely available admission laboratory data capture clinically meaningful information for outcome prediction. The present findings are clinically relevant because admission laboratory values are universally available, objective, and obtained as part of standard care. Unlike neurological grading, they do not depend on clinical examination, which may be limited in sedated, intubated, or critically ill patients. This supports their potential role as complementary markers for early risk stratification in the acute setting.

Established clinical severity scores (GCS, WFNS, Hunt & Hess) provided superior discrimination in isolation. However, the delta between lab-only and clinical-only performance was small, suggesting that the laboratory profile captures partially independent prognostic information. The combined model confirmed that lab values provide additive value beyond clinical scores. Given that the clinical course following aSAH depends on various factors, such as age, severity of bleeding, and comorbidities, its prediction remains a complex and critical challenge in clinical practice (Lovelock et al., 2010). The present data is in line with other published data including the Hunt & Hess grade and the WFNS grade to evaluate the outcome following SAH (Rosengart et al., 2007). For example, a recent model by *Mourelo* et al. predicting in-hospital mortality in patients with aSAH identified AUCs ranging around 80% (Mourelo-Fariña et al., 2021). However, given that no laboratory values on admission were included, one may speculate that the inclusion of admission values, especially glucose, may further enhance the performance of that model. From a translational perspective, such models could be integrated into electronic health record systems to provide automated, real-time risk estimation at admission. Given its interpretability and stable performance, interpretable models such as logistic regression may facilitate future clinical translation after external validation. The present comparison of algorithms also points on a broader methodological question: whether machine learning offers advantages over traditional regression for prediction of outcome after aSAH. In the present data, the linear logistic-regression model performed comparably to the more complex algorithms for the clinical and laboratory feature sets and only in the combined, higher-dimensional setting did ML methods (random forest) yield an advantage. This pattern is consistent with the literature. A systematic review of over 70 comparisons found no overall performance benefit of ML over logistic regression for clinical prediction (Christodoulou et al., 2019). ML algorithms are comparatively data-hungry and require substantially larger samples to realize their theoretical advantage (van der Ploeg et al., 2014) and in neurological prognostication specifically, machine learning performed no better than regression models in traumatic brain injury (Gravesteijn et al., 2020) and only comparably to established clinico-radiological scores in aSAH (Dengler et al., 2021). Advantages of ML methods therefore emerge mainly where non-linearities and higher-order interactions are present, rather than where a handful of strong, largely additive predictors dominate. Consistent with this, the incremental value of laboratory data was captured by the random forest but not by logistic regression in the SAHIT-benchmark analysis, underscoring that any gain from routine laboratories is realized through interaction structure rather than additive linear effects. For clinical translation, however, interpretability and calibration matter at least as much as discrimination, and poor calibration has been termed the “achilles heel” of predictive analytics (Van Calster et al., 2019). Moreover, the modest absolute performance gains argue against replacing transparent clinical scores with complex models in the absence of external validation.

The magnitude of the incremental gain also has implications for cost-effective use of laboratory testing. Most of the laboratory signal was carried by inexpensive, universally obtained parameters: The complete blood count, CRP, glucose, creatinine, and coagulation. Selectively ordered, more costly cardiac and metabolic assays, which also had the highest missingness, contributed little and could be omitted without loss of discrimination, as shown in the sensitivity analyses. From a resource utilization perspective, this suggests that a routinely available admission panel captures relevant prognostic information and that extensive blood work ordered primarily for prediction of outcome is unlikely to be cost-effective.

This study has several limitations. First, the retrospective single-center design and the absence of external validation limit generalizability; the sequential-entry hold-out analysis provides only internal, temporally ordered validation and does not substitute for an independent cohort. Second, several laboratory parameters had substantial missingness. Because extended panels are ordered on clinical suspicion, these data are plausibly missing not at random as patients undergoing cardiac or metabolic work-up may differ systematically from those with only basic laboratories, so median imputation could bias effect estimates. The reassuringly stable performance when high-missingness markers were excluded mitigates, but does not eliminate, this concern. Third, hyperparameters were fixed rather than tuned, which yields conservative but potentially suboptimal performance. Fourth, the data were collected between 2009 and 2015 and subsequent evolution of aSAH management may limit the currency of the absolute risk estimates, although the identified predictors are pathophysiologically stable. In addition, the SAHIT-type model used for benchmarking only approximates the published SAHIT model. Premorbid hypertension, a component of the SAHIT core model, was not recorded in our database and the reference model was re-fitted within the present cohort rather than applied with its published coefficients. The benchmark therefore quantifies the incremental value of laboratory data over SAHIT-type predictors and does not represent an external validation of SAHIT. Because re-fitting favours the reference model, the incremental value is estimated conservatively. Finally, the modest sample size constrains the complexity of models that can be reliably fitted and widens the confidence intervals around between-model comparisons. Future work should validate these models in independent, multicenter and prospective cohorts, incorporate admission laboratories into established scores such as SAHIT, and evaluate whether recalibrated, interpretable models embedded in electronic health records can support real-time risk estimation at admission.

## 5 Conclusion

Routinely obtained admission laboratory parameters carry modest but statistically significant incremental prognostic information beyond established clinical grading after aSAH realized primarily through non-linear machine-learning models and driven by inexpensive, universally available markers such as RDW, glucose, and CRP. Laboratory data did not match clinical grading as a stand-alone predictor and should be regarded as complementary rather than as a replacement for clinical assessment. Given the single-center design, overlapping between-model confidence intervals and the absence of external validation, these results are hypothesis-generating and require prospective, externally validated confirmation before clinical application.

## Statement and declarations

The authors have nothing to declare and there is no potential conflict.

## Conflict of interest

We confirm that the manuscript complies with all instructions to authors, further we confirm that the authorship requirements have been met and the final manuscript was approved by all authors. We confirm that this manuscript has not been published elsewhere and is not under consideration by another journal. Lead ethics approval was approved and the study was performed according to the Declaration of Helsinki. There is no conflict of interest of any of the authors. The study received no funding
