# Supplementary material for: Admission laboratory parameters for early risk stratification after aneurysmal subarachnoid hemorrhage: a machine learning analysis of real-world data
Source: Brain Spine. 2026 Aug 7;6:106270. doi: 10.1016/j.bas.2026.106270 (PMC13485615; doi:10.1016/j.bas.2026.106270)
Supplement: Supplementary file 1 [file mmc1.docx]

**Supplementary Table S1. Sensitivity analyses for missing values.** Abbreviations: AUC = Area under the curve, CI = Confidence Interval

| **Analysis** | **Logistic Regression**  **AUC (95%-CI)** | **Random Forest**  **AUC (95%-CI)** | **SVM**  **AUC (95%-CI)** |
| --- | --- | --- | --- |
| Laboratory set:  all 21 parameters | 0.694 (0.641-0.744) | 0.724 (0.670-0.772) | 0.714 (0.659-0.766) |
| Laboratory set:  Parameters <50% missing (n=17) | 0.698 (0.645-0.749) | 0.710 (0.656-0.758) | 0.708 (0.655-0.760) |
| Laboratory set:  parameters <30% missing (n=15) | 0.665 (0.610-0.717) | 0.652 (0.595-0.705) | 0.662 (0.607-0.717) |
| Combined set:  all parameters | 0.760 (0.708-0.807) | 0.799 (0.753-0.842) | 0.779 (0.730-0.824) |
| Combined set  labs <50% missing | 0.771 (0.720-0.817) | 0.802 (0.756-0.843) | 0.770 (0.719-0.815) |
| Combined set:  labs <30% missing | 0.776 (0.726-0.822) | 0.800 (0.753-0.842) | 0.763 (0.712-0.809) |
