# Supplementary material for: Admission laboratory parameters for early risk stratification after aneurysmal subarachnoid hemorrhage: a machine learning analysis of real-world data
Source: Brain Spine. 2026 Aug 7;6:106270. doi: 10.1016/j.bas.2026.106270 (PMC13485615; doi:10.1016/j.bas.2026.106270)
Supplement: Supplementary file 2 [file mmc2.docx]

**Supplementary Table S2. Sequential-entry analysis.** Models trained on the first 70% of consecutively enrolled patients (n=272) and tested on the last 30% (n=117, 61 unfavorable). Abbreviations: AUC = Area under the curve, CI = Confidence Interval.

| **Feature set** | **Logistic Regression**  **AUC (95%-CI)** | **Random Forest**  **AUC (95%-CI)** | **SVM**  **AUC (95%-CI)** |
| --- | --- | --- | --- |
| **Laboratory** | 0.662 (0.563-0.759) | 0.720 (0.616-0.809) | 0.677 (0.573-0.774) |
| **Clinical** | 0.721 (0.629-0.811) | 0.675 (0.574-0.775) | 0.735 (0.641-0.826) |
| **Combined** | 0.712 (0.616-0.805) | 0.754 (0.664-0.842) | 0.713 (0.617-0.803) |
