# Supplementary material for: Admission laboratory parameters for early risk stratification after aneurysmal subarachnoid hemorrhage: a machine learning analysis of real-world data
Source: Brain Spine. 2026 Aug 7;6:106270. doi: 10.1016/j.bas.2026.106270 (PMC13485615; doi:10.1016/j.bas.2026.106270)
Supplement: Supplementary file 3 [file mmc3.docx]

**Supplementary Table S3. Benchmarking against a SAHIT-type clinical reference model (without the variable hypertension) and incremental value of admission laboratory parameters (cross-validated AUC, n=389).**

| **Model** | **SAHIT-core reference**  **AUC (95%-CI)** | **SAHIT-core + laboratory**  **AUC (95%-CI)** | **ΔAUC (95% CI)** | **p-value** |
| --- | --- | --- | --- | --- |
| **Logistic Regression** | 0.784 (0.736-0.828) | 0.774 (0.725-0.820 | -0.010  (-0.041-+0.021) | 0.520 |
| **Random Forest** | 0.718 (0.664-0.769) | 0.797 (0.751-0.839) | +0.079  (+0.037-+0.119) | <0.001 |
