# Supplementary material for: Admission laboratory parameters for early risk stratification after aneurysmal subarachnoid hemorrhage: a machine learning analysis of real-world data
Source: Brain Spine. 2026 Aug 7;6:106270. doi: 10.1016/j.bas.2026.106270 (PMC13485615; doi:10.1016/j.bas.2026.106270)
Supplement: Supplementary file 4 [file mmc4.docx]

**Supplementary Table S4. Prediction of in-hospital mortality (n = 389, 107 deaths)**

| **Feature set** | **Model** | **AUC (95% CI)** | **Brier** | **Sensitivity** | **Specificity** | **PPV** | **NPV** |
| --- | --- | --- | --- | --- | --- | --- | --- |
| **Laboratory** | Random Forest | 0.694  (0.634-0.753) | 0.181 | 83.2% | 48.6% | 38.0% | 88.4% |
|  | SVM | 0.686  (0.624-0.743) | 0.182 | 68.2% | 62.1% | 40.6% | 83.7% |
|  | XGBoost | 0.658  (0.594-0.722) | 0.217 | 76.6% | 50.7% | 37.1% | 85.1% |
|  | Logistic Regression | 0.654  (0.588-0.717) | 0.236 | 66.4% | 62.8% | 40.3% | 83.1% |
| **Clinical** | Random Forest | 0.821  (0.773-0.867) | 0.149 | 82.2% | 72.0% | 52.7% | 91.4% |
|  | XGBoost | 0.809  (0.761-0.854) | 0.168 | 83.2% | 67.4% | 49.2% | 91.3% |
|  | Logistic Regression | 0.807  (0.757-0.853) | 0.181 | 86.9% | 63.8% | 47.7% | 92.8% |
|  | SVM | 0.803  (0.754-0.849) | 0.152 | 84.1% | 69.9% | 51.4% | 92.1% |
| **Radiographic** | Logistic Regression | 0.729  (0.672-0.780) | 0.215 | 74.8% | 64.5% | 44.4% | 87.1% |
|  | Random Forest | 0.681  (0.624-0.736) | 0.213 | 78.5% | 54.3% | 39.4% | 86.9% |
|  | XGBoost | 0.679  (0.624-0.733) | 0.193 | 79.4% | 51.4% | 38.3% | 86.8% |
|  | SVM | 0.671  (0.611-0.725) | 0.184 | 85.0% | 49.3% | 38.9% | 89.7% |
| **Combined** | Random Forest | 0.828  (0.780-0.871) | 0.147 | 79.4% | 72.0% | 51.8% | 90.2% |
|  | XGBoost | 0.812  (0.763-0.857) | 0.161 | 72.0% | 78.0% | 55.4% | 88.0% |
|  | SVM | 0.792  (0.741-0.837) | 0.158 | 89.7% | 61.0% | 46.6% | 94.0% |
|  | Logistic Regression | 0.763  (0.708-0.811) | 0.201 | 82.2% | 68.8% | 50.0% | 91.1% |
